# Biosynthesis, Physicochemical Characterization, and Functional Evaluation of Chitosan from *Ganoderma lucidum* for the Inhibition of Apple Brown Rot Caused by *Monilinia fructicola*

**DOI:** 10.3390/molecules31162754

**Published:** 2026-08-07

**Authors:** Hazem S. Elshafie, Amira A. Mohamed, Ippolito Camele

**Affiliations:** 1 Department of Agricultural, Forestry, Food and Environmental Sciences (DAFE), University of Basilicata, 85100 Potenza, Italy; hazem.elshafie@unibas.it; 2 Department of Basic Science, Zagazig Higher Institute of Engineering and Technology, Zagazig 44519, Egypt; aa.adaim@science.zu.edu.eg

**Keywords:** biopolymer, bioactive polysaccharides, antimicrobial agents, natural products, postharvest decay, membrane leakage

## Abstract

Chitosan, a chitin-derived biopolymer, is widely used in agriculture and food preservation. Fungal-derived chitosan offers a sustainable alternative to crustacean sources, and *Ganoderma lucidum* represents a promising source of this functional material. This study characterized fungal chitosan (F.Cs) extracted from *G. lucidum* cell-wall chitin by FT-IR, molecular weight, viscosity, and degree of deacetylation. Its antimicrobial activity was evaluated in vitro, and its preservative and disease control efficacy against *Monilinia fructicola* on postharvest apples was compared to commercial chitosan (C.Cs). DNA/protein leakage of the studied FGs and C.Cs were investigated to reveal the antimicrobial mechanisms. In particular, F.Cs exhibited promising in vitro antimicrobial activity, with MIC/MFC values of 0.75/0.75 mg/mL against *M. fructicola* and 0.75/1.5 mg/mL against *Botrytis cinerea*, compared to C.Cs. In addition, F.Cs induced greater membrane damage in *M. fructicola* than C.Cs, as evidenced by higher DNA leakage (24% vs. 13%) and more pronounced protein leakage (102% vs. 98%). In vivo assay demonstrated that F.Cs formed an effective protective coating layer, reducing fruit moisture loss, preserving firmness s, and significantly limiting *M. fructicola*-induced decay compared to C.Cs. Particularly, F.Cs (1.5%) were most effective, reducing disease incidence to 12.5% (preventive) and 20.8% (curative), with decay rates of 18 and 31%, respectively. C.Cs (1.5%) provided moderate protection, with disease incidences of 37.5 and 43.7% in preventive and curative treatments, respectively. These results demonstrate the superior efficacy of F.Cs over C.Cs in preserving apple quality and enhancing resistance to *M. fructicola*, highlighting the potential of *G. lucidum*-derived chitosan as a sustainable, multifunctional biopolymer for postharvest disease control and fruit quality preservation.

## 1. Introduction

Chitin is one of the most common biopolymers in nature [1]. The structure of chitin (C_8_H_13_O_5_N)_n_ consists of 2-acetamido-2-deoxy-β-D-glucose (NAG) monomer units linked via β(1→4) linkages. Chitosan is the deacetylated form of chitin and is soluble in acidic solutions [2]. The conversion of chitin to chitosan can be achieved either by enzymatic methods or chemical hydrolysis [3].

Chitin and chitosan have economic importance due to their advantageous features, such as biodegradability, bio-adhesiveness, and non-toxicity [4,5]. In the EU and UK, chitosan is classified as a “basic substance,” and its use is allowed on a variety of crops and postharvest applications as a biological fungicide and bactericide [6,7]. Chitin and chitosan serve a wide range of purposes, including food manufacturing and agricultural practices, water purification, biomedical research, and diverse biotechnological applications [2]. Although chitin molecules reduce digestibility, they provide a beneficial biological role as part of dietary fibers [8].

Chitin is a component of many diverse organisms, including fungal cell walls, certain algae, and the exoskeletons of arthropods, acting as a protective element [9,10]. Chitin has long been traditionally obtained from the cuticles of insects, squid bones, and crab shells [10,11]. In the seafood sector, waste by-products, including the shells of shrimp and crabs are used to produce chitosan [12,13]. Although the chitin and its derivative chitosan extracted from shrimp shells showed bioactivity against foodborne microorganisms, highlighting their potential as a natural antimicrobial alternative [12], the extraction process from crab shells is limited by geographical and seasonal availability, as well as the need for strong solvents and elevated temperatures [14]. Therefore, obtaining chitin from fungal cell walls has recently become an important sustainable source. This biotechnology offers advantages in producing chitin and chitosan from fungal mycelium, which has gained extensive attention recently due to several benefits. Among them, fungal chitin can be produced in a controlled environment, avoiding unfavorable climatic and geographical conditions. Additionally, the extraction process of fungal chitin is simpler and requires fewer organic solvents than conventional methods [15,16]. Furthermore, fungal mycelia contain lower levels of inorganic materials compared to crustacean shells, and no demineralization treatment is required during processing [17].

Some studies reported that chitin can be extracted from several fungi, such as *Penicillium* spp. [18], *Aspergillus fumigatus* [19], *Trichoderma* spp. [20], *Termitomyces titanicus* [21], *Candida albicans* [22], *Ganoderma lucidum* [23], *Agaricus bisporus* [24], *Pleurotus ostreatus* [24] and *P. eryngii* [9]. Vetter [24] analyzed the chitin contents of pilei and stipes of fruit bodies of some edible mushrooms, such as *Agaricus bisporus*, *Pleurotus ostreatus*, and *Lentinula edodes*, and concluded that its content is a characteristic of the species and seems to be independent of the varieties. Additionally, the results showed that the chitin of the studied cultivated mushrooms is not only a stable structural component of the fungal cell wall but also plays an important role in the nutritional value of mushrooms.

The large amount of mushroom waste is not just a disposal problem, but it is also a valuable resource. Fungal chitosan can be utilized as biopesticides [1,25], antimicrobial agents [26,27], coating materials [28] and for water purification [29]. In the last thirty years, global mushroom production has increased from 13.8 to 42.8 million tons [30,31]. Consequently, fungal waste has also increased annually, representing a real threat to the environment and creating high economic costs for its disposal [16].

One of the most promising recent strategies for managing plant diseases is the application of natural antimicrobial agents for postharvest crop protection. Among the most damaging pathogens affecting apples is *Monilinia fructicola* (G. Winter) Honey, the causal agent of brown rot, also known as blossom wilt [32]. This fungus is responsible for fruit decay, blossom blight, and cankers on young shoots, causing substantial economic losses during both production and postharvest storage [33]. In Italy, brown rot is a widespread disease of apples and is associated with *M. fructicola*, *M. laxa*, and *M. fructigena* [34].

The biological performance of fungal-derived chitosan is related to its physicochemical properties, such as molecular weight, degree of deacetylation, viscosity and also its structure. These characteristics can affect its solubility and charge density, which in turn determine its possible interactions with microbial cells and hence affect its antimicrobial efficiency.

To the best of our knowledge, this is the first study evaluating *G. lucidum*-derived chitosan for controlling *M. fructicola*-induced brown rot in apple fruit. Previous studies have mainly focused on commercial chitosan or oligochitosan against this pathogen. Therefore, the present research highlights the potential of *G. lucidum* as a sustainable fungal source of chitosan for postharvest protection, integrating physicochemical characterization, antifungal evaluation, fruit preservation assessment, comparison with commercial chitosan, and the investigation of antimicrobial mechanisms through DNA/protein leakage analysis.

The main specific objectives of this study are to (i) extract chitin from the cell wall of *G. lucidum* (Curtis) P. Karst., and convert it into chitosan; (ii) conduct a comprehensive physicochemical characterization of the resulting chitosan using Fourier Transform Infrared Spectroscopy (FT-IR), molecular weight (M.Wt) determination, viscosity measurements, and assessment of the degree of deacetylation (DD%); (iii) evaluate the in vitro antifungal activity against three phytopathogenic fungi; (iv) investigate in vivo antifungal efficacy in a postharvest assay against *M. fructicola*, the causal agent of apple brown rot and preservative effect on tested fruits; (v) and estimate the effect of studied chitosan on the DNA/protein leakage of *M. fructicola*.

## 2. Results

### 2.1. Quantification of Biomass, Chitin, and Chitosan

The results of fungal biomass, extracted chitin, and deacetylated chitin “chitosan” are listed in Table 1. The values of chitin and chitosan yields are expressed as percentage of dry weight (g). In particular, the fungal culture in PDB produced 55.93 g of fresh mycelium, corresponding to 11.80 g of dry biomass, where the yield is equal to 21.1%. From 11.8 g of dry biomass, about 1.97 g of chitin was extracted, where the yield is equal to 16.7% (*w*/*w*). Meanwhile, the final obtained chitosan was 0.43 g, corresponding to a yield percentage equal to 3.6% (*w*/*w*) on a dry biomass basis.

### 2.2. Physicochemical Characteristics of Chitin and Chitosan

#### 2.2.1. Infrared Spectra

**Figure 1:**
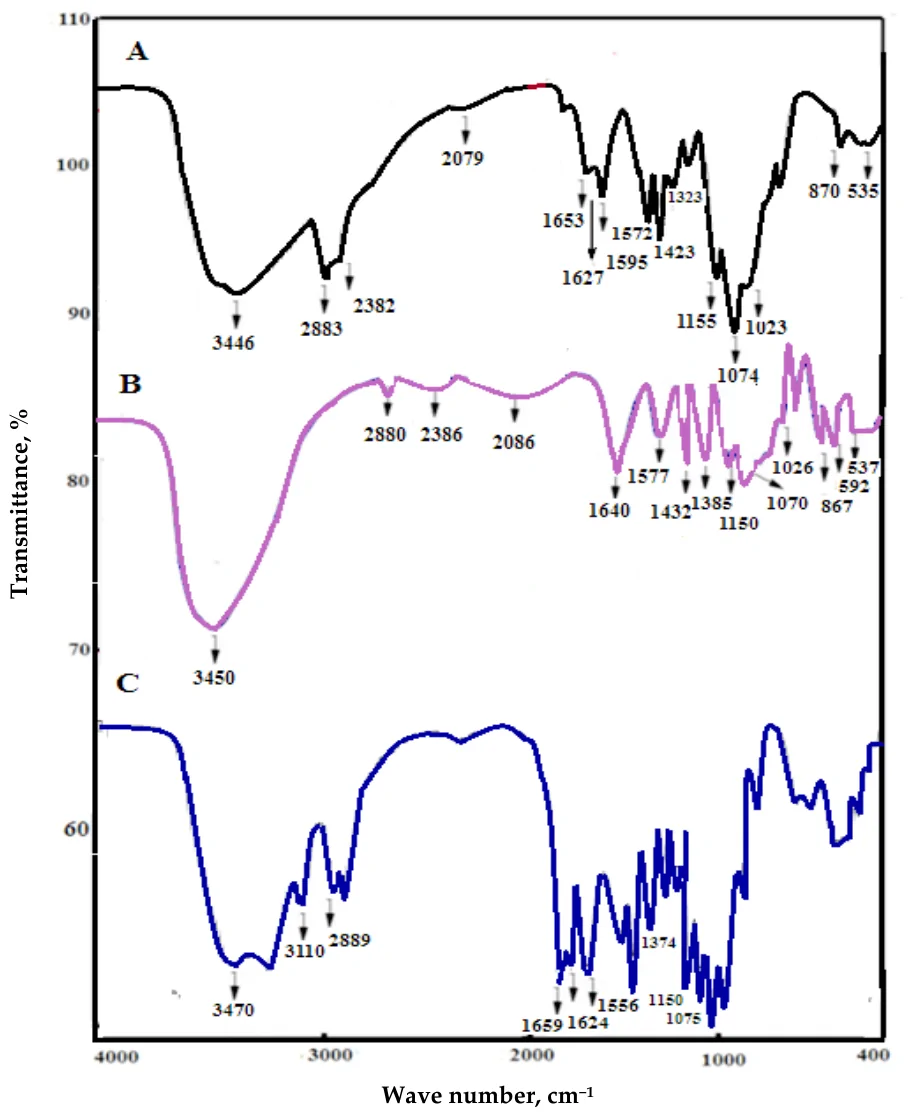
FT-IR spectra of A: commercial chitosan (C.Cs), B: fungal chitosan (F.Cs), and C: chitin (Ct).

Results of the investigation, which are displayed in Figure 1, revealed the presence of several bands between 4000 and 400 cm^−1^. F.Cs, C.Cs, and Ct constitute the FT-IR spectrum. The IR spectra, which were quite similar to crude Cs, demonstrated that the F.C structure remains stable during the depolymerization procedure [35,36]. The FT-IR spectra of the F.Cs under study are shown in Table 2 compared to Ct and C.Cs. Specifically, the presence of an OH group is indicated by a band present at 3450 cm^−1^ [37,38]. The band 2883 cm^−1^ corresponds to C-H stretching. In addition, the presence of the C=O group is shown by a band at around 1650 cm^−1^. There was the presence of a band around 1577 cm^−1^ related to the N-H bending group in the F.Cs, which shifted to 1572, and 1556 cm^−1^ in C.Cs and Ct, respectively. The C-C and C-N groups were identified as the bands at around 1070 cm^−1^ and 1026 cm^−1^, respectively [39,40].

#### 2.2.2. Molecular Weight, Viscosity and Deacetylation Degree

The physical properties of the studied F.Cs are listed in Table 3. The M.Wt of F.Cs was determined using the Mark–Houwink–Sakurada (MHS) equation, $\left[\eta \right]=K\cdot M^{a}$, with constants K=0.078 and a=0.76 for the solute–solvent system (1% acetic acid) at room temperature. The intrinsic viscosity [η] was derived from flow time measurements using Ostwald viscometer (Model AMV-200, Paar Physica, Edison, NJ, USA; inclination angle 15°, capillary diameter 0.9 mm), based on the relation:

(1) $$\eta =(t-t_{0})/t_{0}$$

For a chitosan concentration of 0.5 g/dL in 1% acetic acid, the experimentally obtained [η] ranged from 1.3 to 1.6 cP. Using the measured relative viscosity, the M.Wt of F.Cs was calculated from the intrinsic viscosity value of 1.6 cP via the MHS equation and yielded an M.Wt of $5.3\times 10^{4}$ g/mol equal to 53 kDa.

(2) $$M={\left(\frac{\left[\eta \right]}{K}\right)}^{1/a}$$

where K=0.078 and a=0.76.

The degree of deacetylation (DD%) was determined from the infrared spectra of commercial chitosan (Ct), commercial chitosan (C.Cs), and fungal chitosan (F.Cs), with values of 70.0%, 56.2%, and 63.0%, respectively.

### 2.3. In Vitro Antifungal Activity

Findings from the antifungal test showed that the F.Cs under investigation had a marked inhibitory effect on every fungal pathogen tested. The smallest MIC readings that appeared with F.Cs were 0.75 mg/mL in case of *M. fructicola* and *Botrytis cinerea* compared to 3.0 and 1.5 mg/mL, respectively, in case of C.Cs (Table 4). The MIC of F.Cs against *P. expansum* was 3.0 mg/mL equal to C.Cs. The positive control gave a consistent MIC reading of 100 µg/mL for every fungus in the assay.

When examining fungicidal activity, the minimum fungicidal concentration (MFC) for F.Cs showed the following values: 6.0 mg/mL against *Penicillium expansum*, 0.75 mg/mL against *M. fructicola*, and 1.5 mg/mL against *B. cinerea*. In comparison, C.Cs yielded MFCs of 6.0, 6.0, and 3.0 mg/mL for the same three fungi, respectively (Table 5). Preliminary testing showed that acetic acid at concentrations equivalent to those in the formulations (0.03–0.5%) was ineffective against the tested fungi, likely because it is less effective against the chitin-rich fungal cell wall than bacterial membranes. Thus, antifungal activity can be attributed to F.Cs. However, a synergistic effect cannot be excluded; therefore future studies should evaluate alternative organic solvents at minimal concentrations to exclude eventual solvent-related effects.

### 2.4. In Vivo Fruit-Coating Assay

#### 2.4.1. Quality Parameters

**Figure 2:**
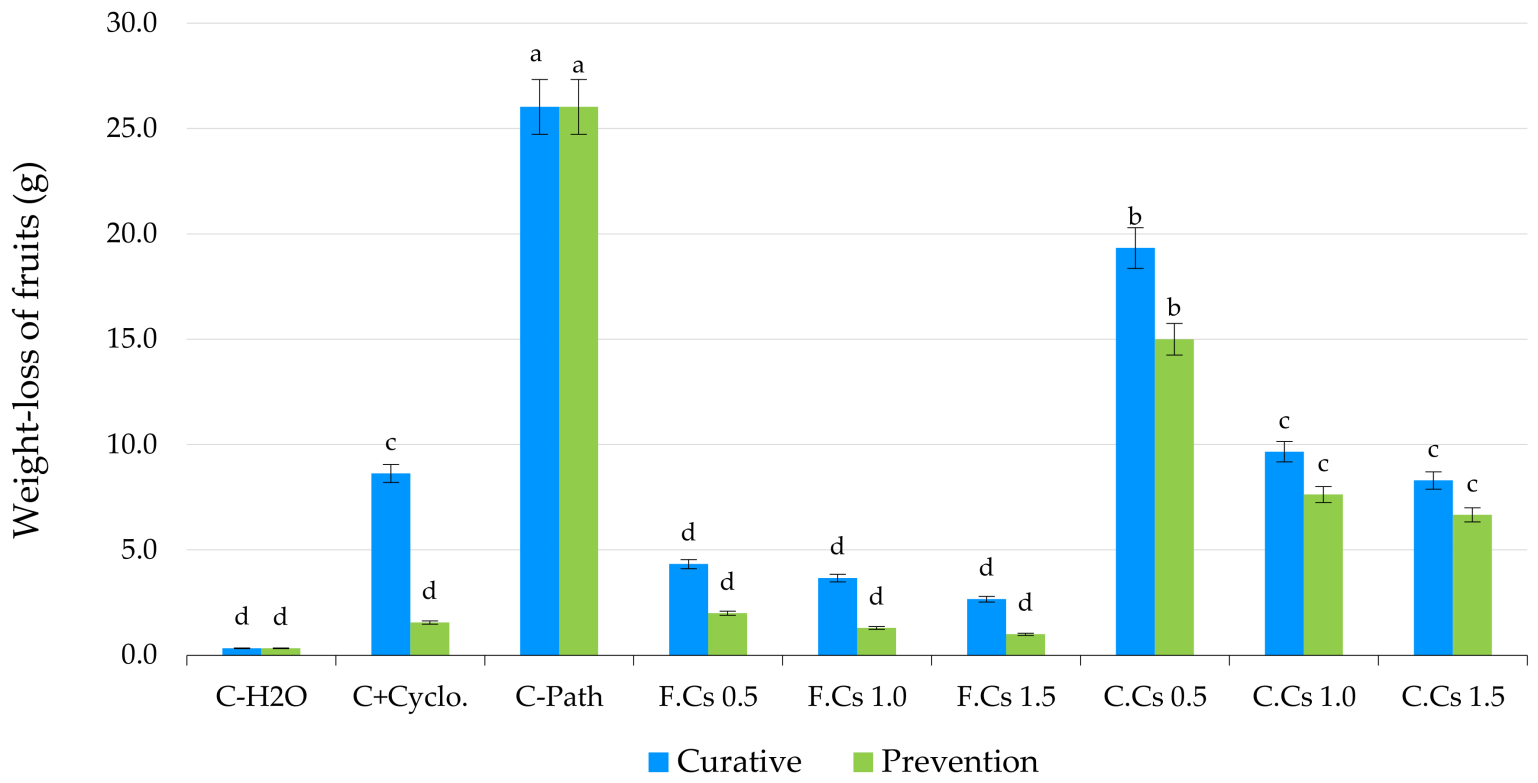
Weight loss of apple measured 14 DAC. C-path: fruits infected only with *M. fructicola*; C+Cyclo: positive control (fruits treated with cycloheximide 100 μg.mL^−1^); C-H_2_O: negative control (untreated fruits). Different letters on bars within the same treatment-model group indicate significant differences (*p* < 0.05) per SPSS. Data are presented as mean ± SD from three replicates per bar.

Weight loss. The results obtained, illustrated in Figure 2, show weight loss measured 14 days after coating (DAC) treatment. The curative model has demonstrated a significantly greater weight reduction in both F.Cs and C.Cs treatments. However, in both models, F.Cs treatments already resulted in minimal weight loss. In conclusion, regardless of the application model, the pathogen control exhibited the greatest weight loss. When compared to prevention, the curative treatment considerably increased the weight loss, particularly with C.Cs treatments, and the F.Cs treatments were quite successful in reducing weight loss in both application models.

**Figure 3:**
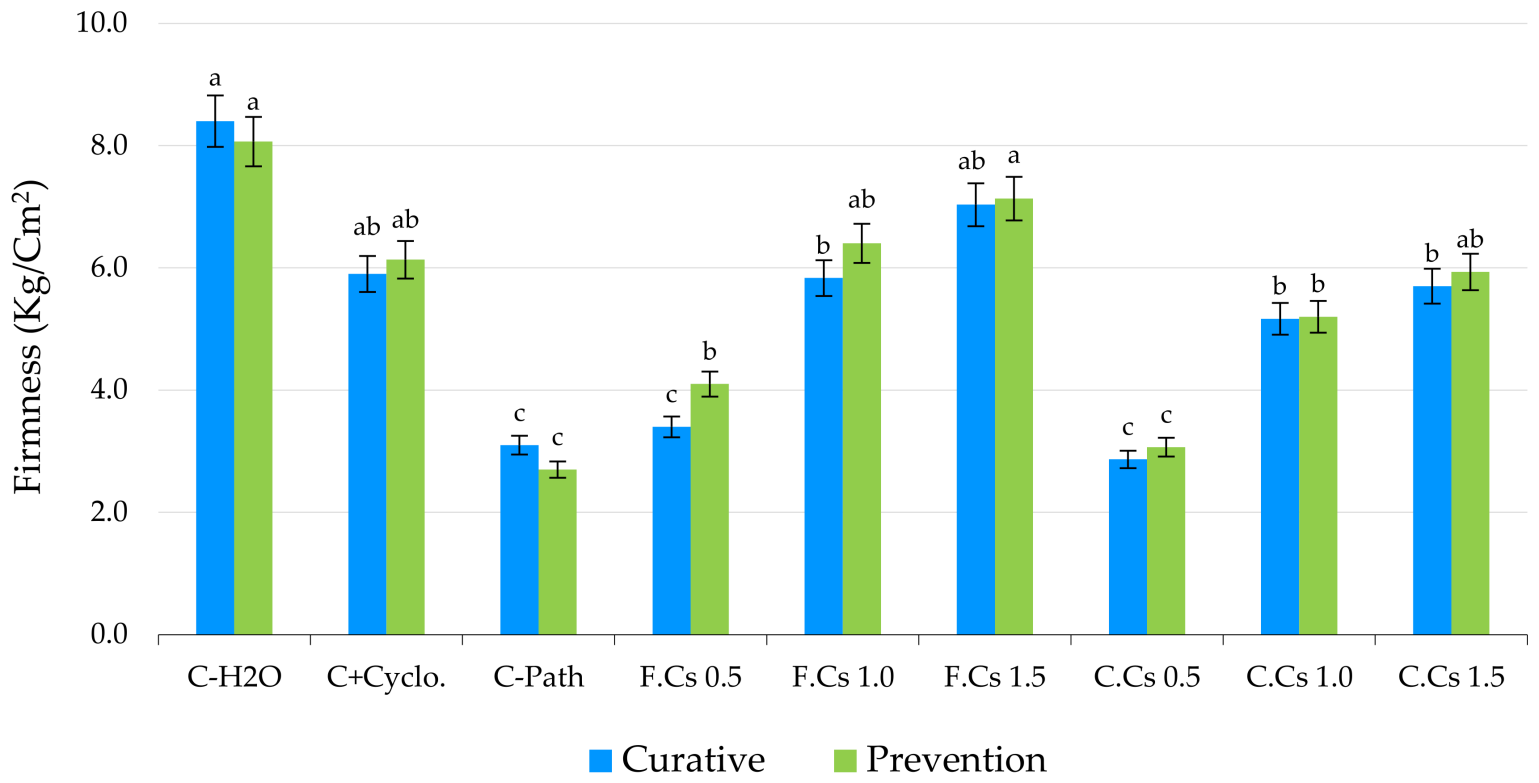
The firmness of apple measured 14 DAC. Different letters on bars within the same treatment-model group indicate significant differences (*p* < 0.05) per SPSS. Data are presented as mean ± SD from three replicates per bar.

Firmness. The obtained results illustrated in Figure 3 of firmness, measured 14 DAC, showed that the prevention model maintains firmness better than the curative model in most F.Cs treatments. In particular, the negative control and F.Cs at 1.5% are able to maintain the highest firmness, which is significantly different from the pathogen control and C.Cs at 0.5%.

**Figure 4:**
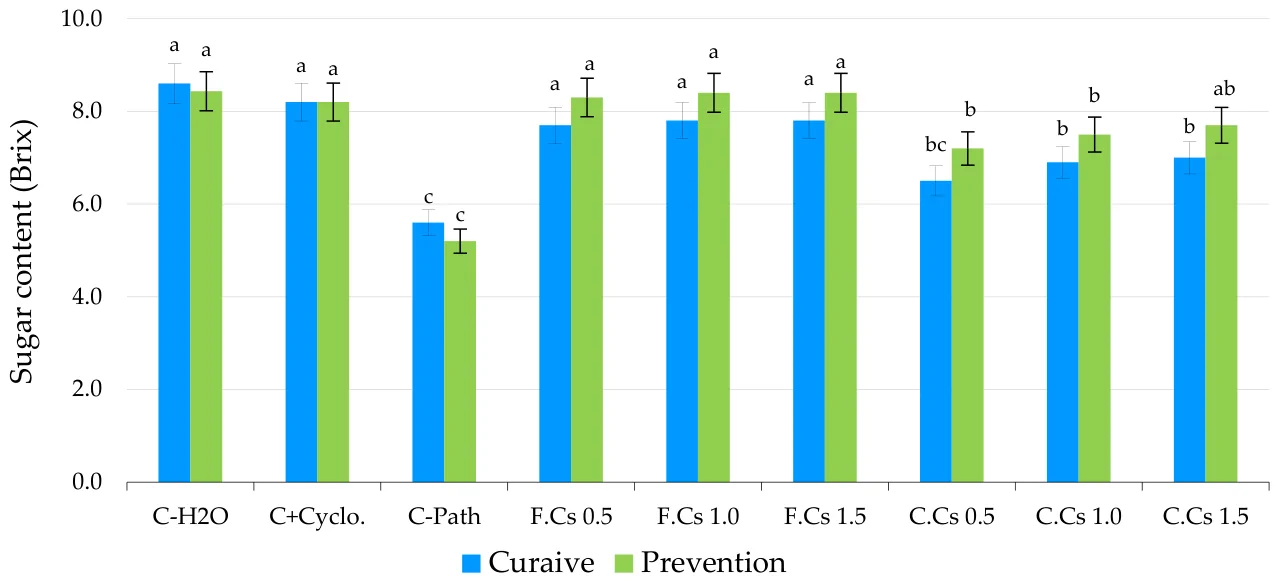
The sugar content of apple measured 14 DAC. Different letters on bars within the same treatment-model group indicate significant differences (*p* < 0.05) per SPSS. Data are presented as mean ± SD from three replicates per bar.

Sugar content. The obtained results illustrated in Figure 4 of sugar content, measured 14 DAC, show that the prevention application has resulted in slightly higher sugar retention compared to curative application for all treatments. In particular, the F.Cs treatments at all tested concentrations are similar to the negative control or even the positive control in both application models, whereas the treatments with C.Cs are less effective but still significantly better. This supports the strategy of applying F.Cs before infection (prevention) to maximize fruit quality preservation and also indicates that F.C is considered a possible natural preservative for sugar content in apples.

**Figure 5:**
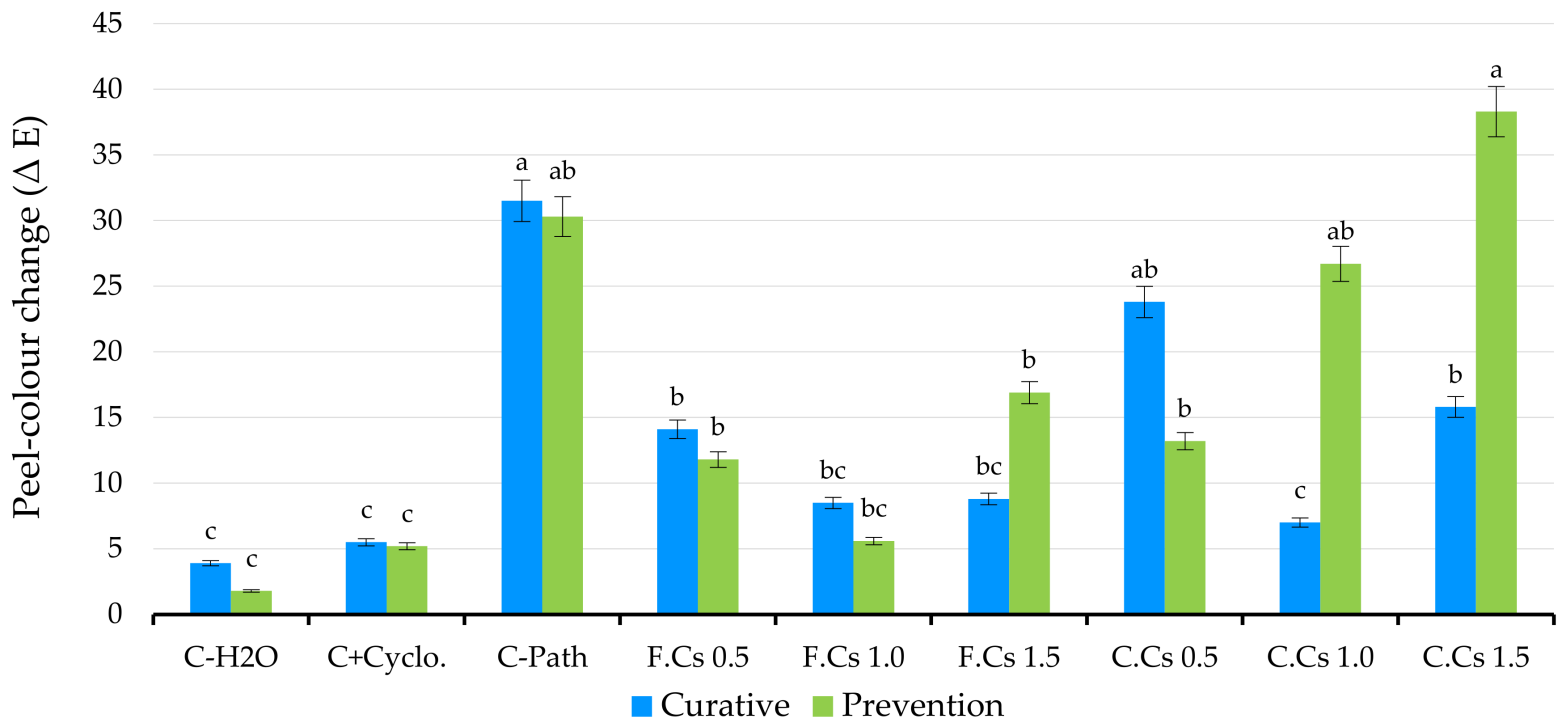
Peel color change in apple measured 14 DAC. Different letters on bars within the same treatment-model group indicate significant differences (*p* < 0.05) per SPSS. Data are presented as mean ± SD from three replicates per bar.

Peel-color. The obtained results illustrated in Figure 5 of peel-color, measured 14 DAC, explicated that the pathogen control leads to extreme color deterioration (ΔE = 31.5) which showed extensive browning, darkening, and loss of visual quality. In particular, the control negative showed a minimal color change over 14 days (ΔE = 1.8). In the prevention model, F.Cs 1% is the most effective treatment (ΔE = 5.6), nearly matching the positive control (5.2) and approaching the healthy control (1.8). Regarding the curative model, the treatments with F.Cs at 1% and 1.5% (ΔE = 8.5–8.8), C.Cs at 1% (ΔE = 7.0) and the positive control (ΔE = 5.5) were able to limit the browning and maintain peel appearance. In addition, the treatment with F.Cs 0.5% showed a moderate peel-color change (ΔE = 14.1).

These results indicated that the application of F.Cs has interesting color-protective properties when preventatively applied. Moreover, the prevention application with C.Cs at 1.5% may cause color deterioration and this explains why the C.Cs may induce browning or stress responses in apple peel when preventatively applied.

#### 2.4.2. Disease Control Effect

**Figure 6:**
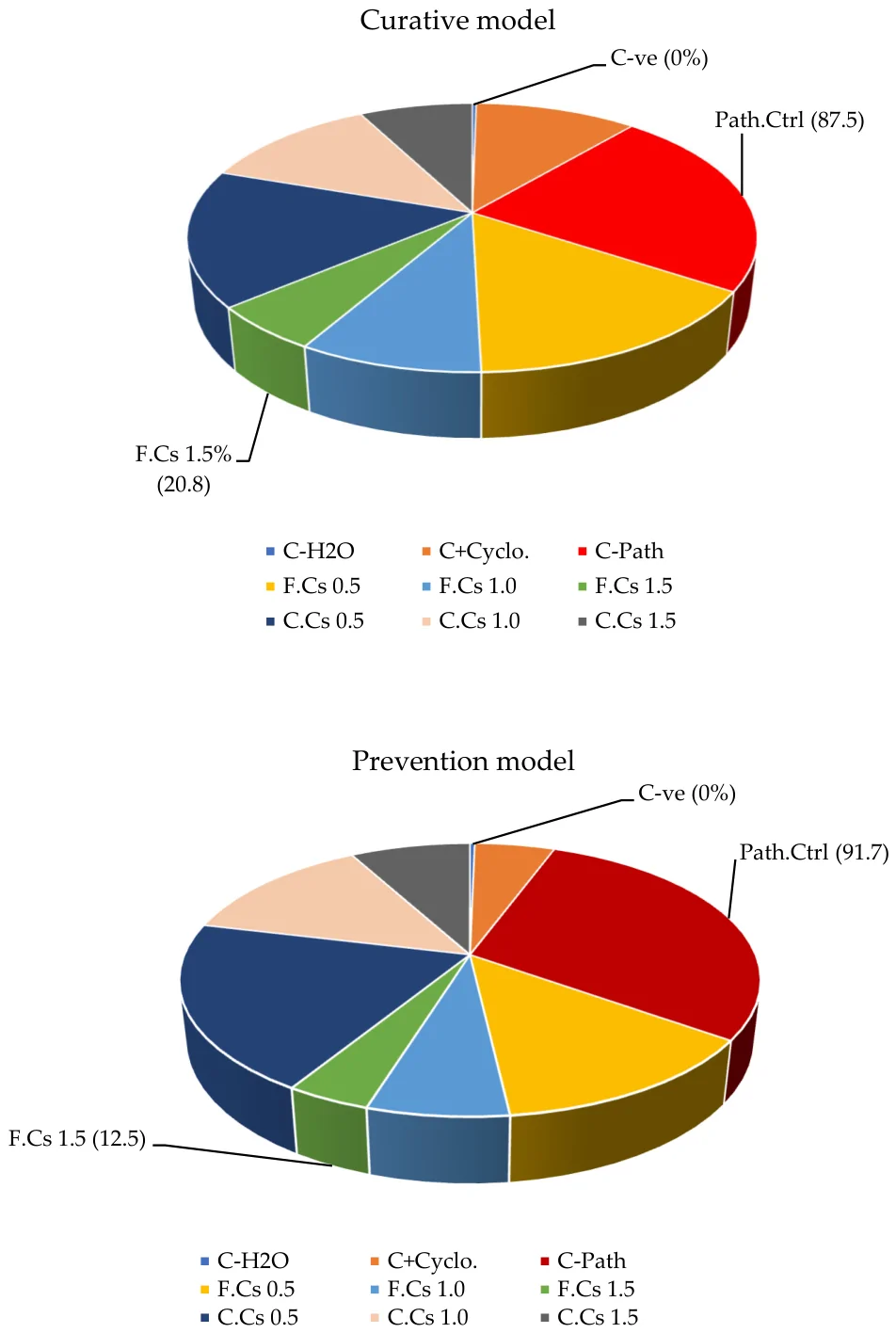
Disease incidence percentage on apple fruits.

Disease incidence. The obtained result of the disease control effect, illustrated in Figure 6, explained that the prevention model has significantly reduced the disease incidence compared to the curative model for most of treatments (Table 6). In particular, F.Cs 1.5% was the most effective treatment for both prevention and curative models, equal to 12.5% and 20.8%, respectively, insignificantly with the synthetic fungicide. Normally, the C-Path showed very high incidence (91.7 and 87.5%) for prevention and curative models, respectively.

**Figure 7:**
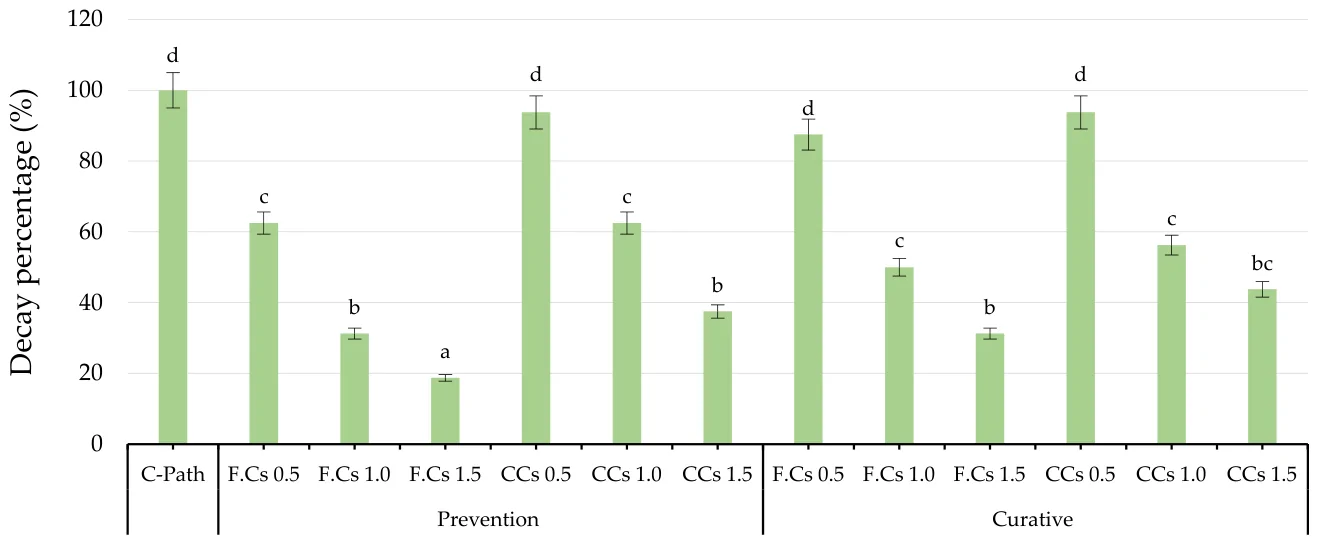
Decay percentage of brown-rot disease on apple fruits either with curative model or prevention model. Different letters on bars within the same treatment-model group indicate significant differences (*p* < 0.05) per SPSS. Data are presented as mean ± SD from three replicates per bar.

Decay percentage. Figure 7 showed the obtained results for the decay percentage due to pathogen infection. The C-Path showed 100% decay, whereas the F.Cs 1.5% was the most effective treatment especially in the prevention model (18.7% decay). In addition, the use of C.Cs at the highest concentration (1.5%) was the only effective one, showing a moderate protection for both prevention and curative models equal to 37.5 and 43.7%, respectively. These findings indicated that the prevention application is better than the curative one for the F.Cs treatments. The application of acetic acid alone on tested fruits, at concentrations equivalent to tested formulations (0.03–0.5%), did not significantly affect disease incidence or decay development and its role in disease suppression was considered minimal. Although the contribution of acetic acid alone appeared negligible under the tested conditions, a possible synergistic or additive effect within the chitosan–acetic acid system cannot be completely excluded. Future studies should therefore investigate alternative organic solvents at minimal concentrations to further clarify and minimize any solvent-collateral effect.

### 2.5. DNA/Protein Leakage (%)

**Figure 8:**
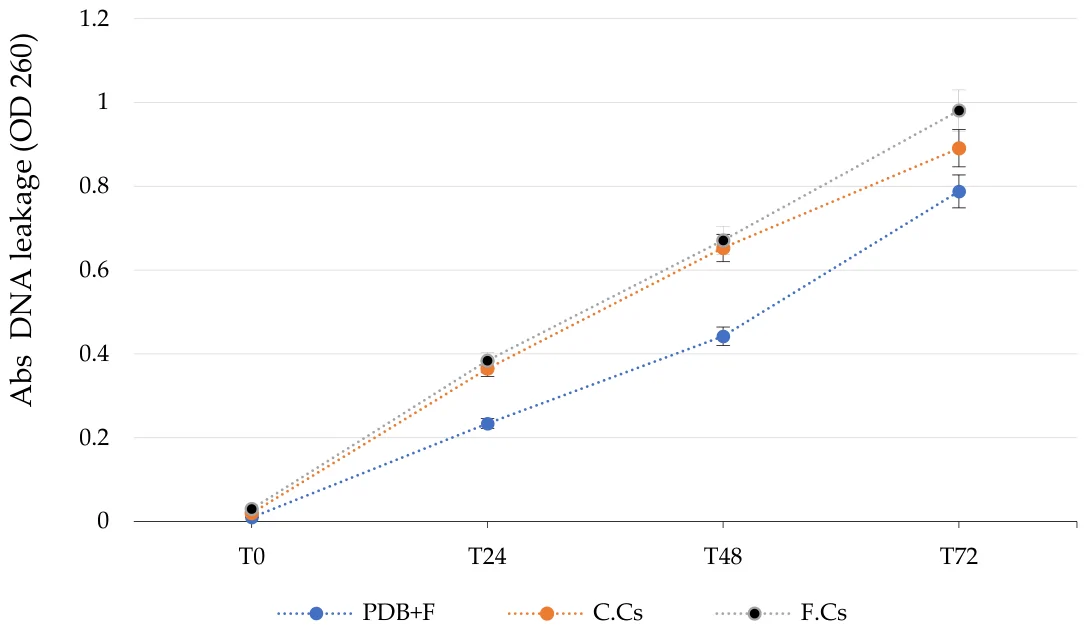
DNA leakage after treatment with fungal and commercial chitosan.

**Figure 9:**
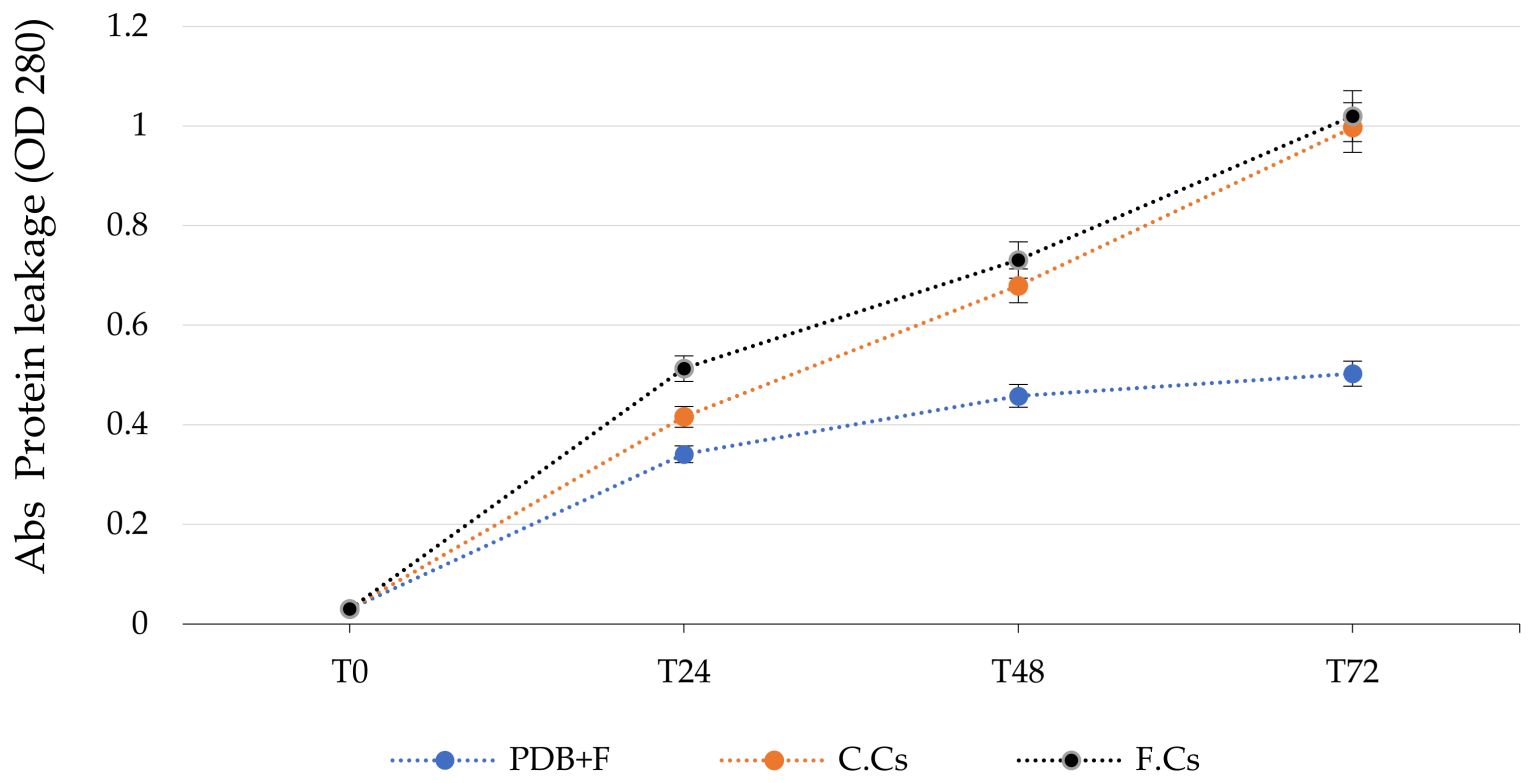
Protein leakage after treatment with fungal and commercial chitosan.

Results illustrated in Figure 8 and Figure 9 showed the protein and DNA leakage after treatment with fungal and commercial. Generally, both C.Cs and F.Cs at 1.5% concentration induced substantial membrane damage in *M. fructicola*, as evidenced by time-dependent increases in extracellular DNA and protein absorbance.

A pronounced disparity was observed between the two leakage markers. The net protein (ΔA280) and DNA (ΔA260) leakage values, presented in Table 7 and Table 8, respectively, were calculated after subtracting the corresponding T0 control (background) values. Protein leakage increased markedly, reaching near-complete levels after 72 h of treatment (98% for C.Cs and 102% for F.Cs). In contrast, DNA leakage remained comparatively low, reaching only 13% for C.Cs and 24% for F.Cs at the same time point. These findings indicate that treatment with the studied chitosan samples caused substantial membrane permeabilization, resulting in extensive protein efflux while only limited DNA release occurred. This difference indicates that chitosan primarily disrupts the plasma membrane, allowing relatively small intracellular proteins to freely diffuse out, while the nuclear envelope remains largely intact. The control sample (PDB+F) already showed baseline leakage over time, likely due to natural autolysis or growth-related stress.

Overall, these results demonstrate that both chitosans are potent membrane-permeabilizing agents against *M. fructicola*, with fungal chitosan offering a slight advantage in promoting nuclear DNA release, which may enhance fungal cell death. The obtained results can explain the effect of chitosan on induction of the cell membrane disruption; however, the nuclear envelope breakdown requires more extensive or prolonged damage compared to protein.

## 3. Discussion

Our results are consistent with previous reports on fungal-derived chitosan. For example, Pochanavanich and Suntornsuk [41] investigated chitosan production from several fungal species, including *Aspergillus niger*, *Rhizopus oryzae*, *Lentinus edodes*, *Pleurotus sajor-caju*, *Zygosaccharomyces rouxii*, and *Candida albicans* and evaluated both biomass production and chitosan yield. They reported that a maximum yield of 130 mg of chitosan per gram of fungal biomass. Likewise, Ospina et al. [42] reported that *G. lucidum* produced approximately 80 mg of chitosan per gram of fungal biomass, corresponding to a yield of 6–10% (*w*/*w*), with moisture contents ranging between 90 and 96 comparisons demonstrate that the extraction process used in the current study is efficient and comparable to those reported for other fungal species.

The extracted fungal chitosan from *G. lucidum* exhibited a relatively low M.Wt (53 kDa) with low intrinsic viscosity (1.3–1.6 cP), both of which are important determinants of its biological activity. Low M.Wt generally exhibits improved solubility and enhanced interaction with negatively charged microbial cell membranes. These properties improve the interaction of chitosan with microbial cell membranes, making them more permeable and causing the leakage of cell contents, which eventually leads to cell death. In addition, the low viscosity of F.Cs may facilitate its diffusion through microbial cell walls. These findings are in agree with those of Zheng and Zhu [43], who demonstrated that chitosan with a M.Wt below 305 kDa exhibits concentration-dependent antimicrobial activity. Moreover, the molecular weight obtained in the present study agrees with previous reports indicating that fungal chitosan, among them *Ganoderma* species, typically range from 40 to 100 kDa or 50–100 kDa, respectively [4,44,45,46,47].

The DD% is another key physicochemical property indicating the bioactivity of chitosan. Taşkın et al. [48] reported that DD% determines the abundance of free amino groups along the polysaccharide chain, making it one of the most important factors influencing chitosan bioactivity. In general, a higher DD% enhances chitosan solubility under acidic conditions. In this regard, Tolaimate et al. [49] demonstrated that chitosan with a higher DD% carries a greater positive charge, resulting in enhanced antimicrobial activity compared to chitosan with lower DD% values. As for overall physical properties, we underline that the combination of low M.Wt (53 kDa), moderate deacetylation (63%), and low intrinsic viscosity (1.3–1.6 cP) acts synergistically to improve the solubility, membrane-binding capacity, and cell-wall penetration of fungal chitosan. However, the M.Wt and DD% are considered the primary determinants.

The antimicrobial activity of chitosan against phytopathogens is mainly attributed to its ability to disrupt microbial cell membranes, increasing membrane permeability and causing the leakage of intracellular components, which ultimately leads to cell death [50]. In addition to membrane disruption, the antifungal activity of chitosan may involve additional mechanisms, including the chelation of essential nutrients and metal ions, interference with DNA and RNA synthesis, and the subsequent inhibition of protein production and cellular metabolism [47,51].

Other beneficial effects of chitosan include the reduction in nematode infection in plant roots, thereby enhancing the natural suppressiveness of soils against plant diseases. In addition, chitosan has been reported to stimulate the activity of beneficial rhizosphere microorganisms, including *Bacillus*, *Pseudomonas*, actinomycetes, rhizobacteria, and mycorrhiza [52,53]. Consistent with our findings, Tayel et al. [54] and Bento et al. [55] reported that low-molecular-weight chitosan extracted from *Mucor rouxii* exhibited strong antimicrobial activity against several bacterial species, including *Staphylococcus aureus*, *Sarcina lutea*, and *Listeria monocytogenes*.

The in vivo results confirmed the effectiveness of chitosan, particularly F.Cs, for controlling *M. fructicola*. Chitosan forms a thin, semi-permeable edible coating on the fruit surface that reduces respiration and water loss, thereby maintaining firmness, color, and soluble sugar content and limiting weight loss, thus extending shelf-life. Our findings are in agreement with those of Rajestary et al. [56], who demonstrated that fungal-derived chitosan effectively controlled major postharvest pathogens, including *Botrytis cinerea*, *Penicillium* spp., and *Colletotrichum* spp., while preserving fruit quality. In our previous study, Camele et al. [9] showed the chitosan extracted from *Pleurotus eryngii* has significantly reduced plum brown rot caused by *Monilinia laxa* and maintained fruit firmness during storage.

Regarding DNA and protein leakage, our results are supported by Ma et al. [57], who demonstrated that chitosan compromises the membrane integrity of *M. fructicola*. They reported that chitosan- and oligochitosan-treated spores exhibited significantly lower plasma membrane integrity than the control, which is consistent with our observation of a time-dependent increase in DNA and protein release following chitosan treatment. Their work thus provides a foundational basis for interpreting our leakage data as direct evidence of chitosan-mediated permeabilization of the plasma membrane in *M. fructicola*.

The possible mechanism of the chitosan action on plasma membranes is discussed also by Palma–Guerrero et al. [58], who reported that there is a biophysical interaction between chitosan and fungal membranes. This mechanism directly explains our obtained results that protein leakage reached near-complete levels (98.2–102.0%) and DNA leakage (13.1–24.5%). Therefore, the main primary action of chitosan on the plasma membrane allows relatively small intracellular proteins to diffuse out freely, whereas the nuclear envelope with double-membrane structure remains largely intact.

## 4. Materials and Methods

### 4.1. Fungal Cultivation and Biomass Quantification

*Ganoderma lucidum* (collection n. 1925), conserved at 4 °C in the mycology collection of the Department of Agricultural, Forestry, Food and Environmental Sciences (DAFE), University of Basilicata, Potenza (Italy), was recultured on Potato Dextrose Agar (PDA) for 96 h at 24 °C. Six pieces squares, each of 5 mm a side, and of PDA with *G. lucidum* mycelium, were used to inoculate three Erlenmeyer flasks (two pieces per flask) in 200 mL potato dextrose broth (PDB); then, were incubated under agitation (200 rpm, 20 days/28 °C) in a rotary incubator (Heidolph WB 2000, Labexchange, Burladingen, Germany) [59]. After the incubation, the inoculated broth was centrifuged (30,000× *g*/20 min). The harvested biomass was rinsed with deionized water, dried overnight under laminar flow hood, and its dry weight was determined following the protocol described by Botella et al. [23].

### 4.2. Extraction and Deacetylation of Chitin

Chitin was extracted from G. lucidum cell wall following the alkaline–acid protocol described by Hahn et al. [60] and El-Far et al. [61] with minor modifications. The extraction process involved three sequential steps: (i) demineralization, (ii) deproteinization, and (iii) deacetylation of chitin to obtain chitosan (Cs). These steps are essential for removing non-chitinous components from fungal biomass and converting the purified chitin polymer into chitosan through the partial removal of acetyl groups.

(i) Demineralization. The fungal biomass was first dehydrated at 60 °C for 48 h and subsequently cryo-milled to a fine powder using Blender (LB20EG, Waring, Stamford CT, USA) to enhance the surface area and facilitate solvent penetration. The powdered biomass was demineralized with 0.5 M formic acid (HCOOH) for 1 h at ambient temperature under continuous magnetic stirring. The biomass was then washed with sterile distilled water until neutral pH (~7.0) to eliminate residual acid.

(ii) Deproteinization. The demineralized biomass was then treated with 2 M sodium hydroxide (NaOH) at 80 °C for 2 h under continuous stirring to facilitate protein removal. The obtained insoluble chitin fraction was recovered by centrifugation and washed with distilled water until neutrality, then dried to constant weight.

(iii) Heterogeneous deacetylation. Chitosan was obtained by treating the dried-demineralized/deproteinized chitin with 12 M NaOH at 100 °C for 2 h under stirring conditions, using a solid-to-liquid ratio of 1.2 mg/mL (60 mg chitin per 50 mL NaOH solution). This alkaline treatment facilitates the cleavage of acetamide groups, converting the polymer to its deacetylated form. After deacetylation, the solid residue was neutralized by washing with distilled water and subsequently dissolved in 1% (*v*/*v*) acetic acid at 40 °C under overnight stirring to obtain a soluble chitosan solution. A commercial chitosan derived from crustacean shells (molecular weight 100–300 kDa; Thermo Scientific, Fair Lawn, NJ, USA) was used as a reference standard for comparative analysis.

Following complete dissolution, the chitosan solution was clarified by filtration or centrifugation to remove any insoluble debris. To minimize residual acetic acid effect, chitosan solutions were adjusted to pH 5.5–6.0 with 1 M NaOH, maintaining solubility while reducing free acid to non-cytotoxic levels.

Before biological testing, chitosan stock solutions were serially two-fold diluted with sterile distilled water, reducing the final acetic acid concentration to 0.03–0.5%. Acetic acid was independently evaluated at the corresponding concentrations to exclude solvent-related effects.

The dry weights of chitin extracted from *G. lucidum* (F.Ct) and its deacetylated derivative “chitosan” (F.Cs) were determined and their yield percentage were calculated as reported by Camele et al. [9].

(3) $$\mathrm{Yield}\;\mathrm{quantity}\;\left(\%\right)=\frac{\mathrm{Dry}\;\mathrm{wt}.\;\mathrm{chitin}/\mathrm{chitosan}\;(g)}{\mathrm{Dry}\;\mathrm{wt}.\;\mathrm{fungal}\;\mathrm{biomass}\;\left(g\right)}\times \;100$$

### 4.3. Physical and Chemical Profiling of Chitin and Chitosan

The following physicochemical analyses have been carried out to the extracted F.Ct and F.Cs.

#### 4.3.1. FT-IR Analysis

To verify the presence of characteristic functional groups in the extracted F.Ct and F.Cs, FT-IR measurements were taken on the more crystalline samples. Spectra were collected with an FT-IR 460 PLUS Spectrophotometer (Shimadzu 8400S, Tokyo, Japan) in the 4000–400 cm^−1^ range, using KBr pellets.

#### 4.3.2. Molecular Weight, Viscosity and Deacetylation Degree

The molecular weight (M.Wt) of F.Cs was determined using the Mark–Houwink–Sakurada (MHS) equation [62], as the viscosity of a polymer solution is directly related to its molecular weight. The MHS equation is expressed as

(4) $$[\eta ]=K\cdot M^{a}$$

where [η] is the intrinsic viscosity, M is the molecular weight, and K and a are constants specific to the solute–solvent system and temperature.

For F.Cs dissolved in 1% acetic acid at the experimental temperature, the constants were taken as K = 0.078 and a = 0.76 [63].

Capillary viscometry with an Ostwald viscometer (Model AMV-200, Paar Physica, Edison, NJ, USA) was used to determine the intrinsic viscosity of F.Cs. To minimize kinetic energy and shear corrections, the instrument was set at a 15-degree tilt and fitted with a 0.9 mm capillary. The flow time of the solvent (1% acetic acid, t_0_) and the flow time of the F.Cs solution (t) were measured using a chronometer, with the solution concentration maintained below 1% (*w*/*v*) as described previously [64,65]. The relative viscosity (η) was calculated according to the following Equation (3).

(5) $$\eta =(t-t_{0})/t_{0}$$

The deacetylation degree (DD%) of F.Cs was evaluated using FT-IR spectroscopy based on the analysis of characteristic absorption bands as described by Brugnerotto et al. [66]. This approach relies on the ratio of absorbance at 1655 cm^−1^ to that at 3450 cm^−1^. The DD% was calculated using the following equation:

(6) $$\mathrm{DD}(\%)=100-\left[\left(\frac{A_{1655}}{A_{3450}}\right)\times 115\right]$$

where $A_{1655}$ corresponds to the absorbance at 1655 cm^−1^, $A_{3450}$ represents the absorbance at 3450 cm^−1^, and the constant value (115) reflects the standard ratio of these absorbance bands.

### 4.4. In Vitro Evaluation of Antifungal Activity

#### 4.4.1. Tested Phytopathogenic Fungi

The antifungal activity was evaluated against the following three phytopathogenic fungi, *Penicillium expansum* Link (CN-1109), *Botrytis cinerea* Pers. (CN-2163), and *Monilinia fructicola* (G. Winter) Honey (CN-1725). These isolates were obtained from the mycology collection located at DAFE department, University of Basilicata (Italy). The studied isolates were sub-cultured prior to use on PDA medium at 24 °C for 96 h.

#### 4.4.2. Minimum Inhibitory Concentration

The minimum inhibitory concentrations (MICs) of F.Cs and commercial chitosan (C.Cs), sourced from crustacean shells with a reported M.Wt between 100,000 and 300,000 Da (Thermo Scientific, Fair Lawn, NJ, USA), were determined against the tested fungal isolates using “Broth Microdilution” assay in 96-microplate (Nunc MaxiSorp^®^, Vedbaek, Denmark) as described by Costa et al. [67]. Potato dextrose broth (PDB) was used for the preparation of fungal suspensions.

The pH of the obtained chitosan was first adjusted to 7.0 to minimize the effect of residual acetic acid. Subsequent serial dilution with sterile distilled water further reduced acetic acid concentration, ensuring that the observed biological effects were primarily attributed to chitosan. A stock solution of F.Cs and C.Cs (6.0 mg/mL) was prepared in 1% (*v*/*v*) acetic acid under continuous stirring and sterilized through a 0.22 µm membrane filter and stored at 4 °C. A series of two-fold serial dilutions (3.0, 1.5, 0.75, 0.375, and 0.187 mg/mL) was prepared in sterile distilled water with final concentration of acetic acid ranged between 0.5 and 0.031%. However, in our previous study, by Camele et al. [9], acetic acid used as a negative control showed no antifungal activity against the tested fungi, either in vitro or in vivo, at the same final concentration employed for chitosan solution preparation.

For the assay, 100 µL/well of each chitosan concentration was dispensed onto the plate. Fungal spores were separated from the mycelium and the resulting spore suspension was adjusted to $1\times 10^{6}$ spores/mL; 50 µL of each fungal suspension was added to each well. The plates were incubated at 24 °C for 48 h. Fungal growth was assessed by measuring absorbance at 590 nm using a microplate reader (DAS s.r.l., Rome, Italy). Wells containing only PDB were used as negative controls. The MIC was defined as the lowest concentration of chitosan that resulted a significant inhibition of fungal growth, as indicated by absorbance values compared to the negative control.

#### 4.4.3. Minimum Fungicidal Concentration

To distinguish between fungistatic and fungicidal effects, the minimum fungicidal concentration (MFC) was determined [68]. Aliquots (50 µL) from wells that showed no visible growth were recultured onto PDA plates and incubated under the same conditions. The MFC was defined as the lowest concentration with no shown fungal growth.

### 4.5. In Vivo Postharvest Assay

This experiment was designed to evaluate the in vivo antimicrobial efficacy of fungal-extracted chitosan from *G. lucidum* on apple fruit artificially infected with *M*. *fructicola* (CN-2547 isolated from apple), which is the causal agent of brown rot. The study has carried out using a completely randomized design including untreated and a positive control.

#### 4.5.1. Fruit Materials

Untreated apple fruit (*Malus domestica*) cultivar Gala, average weight 150–160 g, was used in this trial. The surface sterilization of the fruits was carried out with a 0.5% NaClO, and then they were rinsed twice with sterile distilled water for 3 min to eliminate the residue of the used disinfectant.

#### 4.5.2. Chitosan Treatments

The F.Cs solution was prepared by dissolving the extracted chitosan sample at three concentrations (0.5, 1.0 and 1.5% (*w*/*v*). Glycerol was utilized as a plasticizer at 750 µL/g chitosan, where it can improve the physical properties of the coating and enhances its protective function [69]. The apples were divided into nine groups treated with (i) negative control: fruits treated only with SDW (C-ve); (ii) positive control: fruits treated with cycloheximide at 100 μg/mL (C+ve); (iii) pathogen control: fruits infected with *M. fructicola* (P-Ctrl); (iv) fruits treated with F.Cs at 0.5%; (v) fruits treated with F.Cs at 1.0%; (vi) fruits treated with F.Cs at 1.5%; (vii) fruits treated with C.Cs at 0.5%; (viii) fruits treated with C.Cs at 1.0%; (ix) fruits treated with C.Cs at 1.5%. The fruits were sprayed individually with each treatment solution and subsequently were dried for 30 min under a laminar flow hood for 30 min. Two conceptual treatment models were included: prevention (chitosan applied before pathogen infection) and curative (chitosan applied after pathogen infection).

#### 4.5.3. Quality Parameters Evaluation

Measurements of weight loss, firmness, color variation, and sugar level were taken to assess how the treatment influenced fruit quality after treatment with F.Cs compared to C.Cs. All parameters were detected on five fruits per each treatment at 14 DAC.

Weight loss (%) was measured using the following equation proposed by Kader [70].

(7) $$Weight\;loss\;\left(\%\right)=\frac{Wt.i-Wt.t}{Wt.c}\times 100$$

where Wt.i is the initial weight of fruit, and Wt.t is the weight measured after 14 days.

Firmness was taken by Fruit-Pressure Tester (FT 327, Alfonsine, Italy, Ø = 11 mm). The cylinder was penetrated to 3–5 mm depth of fruit with a speed 2 mm/sec. Firmness was measured and reported in Newtons (N, where 1 N = 1 kg/s^2^), then compared with the negative control values [71].

Color variation was taken on two sides of each fruit using a Colorimeter (Minolta CR 400 Chroma Meter, Minolta Corp., Tokyo, Japan), using the equation proposed by Granato and Masson [72]:

(8) $$\Delta \;E\;=\sqrt{(\Delta L{)}^{2}+\;(\Delta a{)}^{2}\;+\;(\Delta b{)}^{2}\;}$$

where ΔE denotes the overall changes in color indices; L index refers to black-to-white color; a index refers to green-to-red color; b index refers blue-to-yellow color.

Sugar content, a refractometer (HI96800, Hanna, Villafranca Padovana, Italy), provided measurements of the three sugar components: glucose, fructose, and sucrose [73]. The values were determined in degrees Brix, equal to 1 g sucrose/100 g solution.

#### 4.5.4. Artificial Fungal Infection and Decay Evaluation

The fungal strain used for infection was *M. fructicola*, catalog number 1561. Its identity had been confirmed earlier through both morphological traits and molecular data. The isolate’s sequence records are publicly available in the NCBI GenBank under accession number HF678388. This strain is maintained in the DAFE mycology collection. To inoculate the fruits, a spore suspension was adjusted to 10^7^ spores/mL. For the prevention model, the fruits were treated 24 h prior to artificial infection; whereas for the curative model, the fruits were treated 24 h post artificial infection.

Each fruit was wounded using a sterile needle, after which 5 µL of the fungal suspension was placed onto the puncture site following the protocol reported by Camele et al. [9]. The inoculated fruits were then stored at room temperature inside polyethylene plastic bags to maintain humidity. The disease severity index (DSI), lesion diameter (mm) and decay percentage were calculated after 5 days of inoculation following the disease scale, as specified below (Table 9). The experiment was carried out twice, with three replicates.

(9) $$\mathrm{Decay}\;(\%)=\frac{\sum \left(0\times N0+1\times N1+2\times N2+3\times N3\right)}{3\times N}\times 100$$

where N0 = no decay; N1 = slight decay (1–25% of surface decay); N2 = moderate decay (26–50% of surface decay); N3 = severe decay (>50% of surface decay); N = total number of fruits.

### 4.6. Evaluation of DNA/Protein Leakage (%)

Membrane leakage in *M. fructicola* was assessed by measuring the release of intracellular nucleic acids and proteins following chitosan treatment as described [74]. A fungal spore suspension was prepared by inoculating two agar pieces of *M. fructicola* into potato dextrose broth (PDB) and incubating at 25 °C for 48–72 h until active growth was achieved at 10^6^ spores/mL. Four experimental groups were established: (1) fungal suspension only (control), (2) fungus with 1.5% fungal chitosan (F.Cs) extracted from *G. lucidum*, (3) fungus with 1.5% commercial chitosan (C.Cs), and (4) PDB only (control). All groups were incubated at 25 °C with shaking at 120 rpm. At each time point (0, 24, 48, and 72 h), 1 mL aliquots were collected and centrifuged at 8000 rpm for 10 min to eliminate the cells and debris. The resulting supernatant, containing leaked cellular contents, was carefully recovered. Nucleic acid leakage was quantified by measuring absorbance at 260 nm (OD_260_), while protein leakage was determined by absorbance at 280 nm (OD_280_), using a spectrophotometer. Higher absorbance values indicated greater membrane damage and the loss of cellular integrity. The relationship stating that an absorbance of 1.0 at 260 nm (A260) is equal to 50 µg/mL for a pure solution of double-stranded DNA (dsDNA) is a foundational principle in molecular biology [75]. Leakage levels induced by fungal chitosan were compared directly to those induced by commercial chitosan to evaluate relative efficacy. The relative leakage was calculated using the following equation:

(10) $$\mathrm{Relative}\;\mathrm{DNA}/\mathrm{Protein}\;\mathrm{Leakage}\;(\%)=\frac{{OD\;T72}_{t}-{OD\;T72}_{c}}{{OD\;T72}_{c}}\times 100$$

where ${OD\;T72}_{t}$ = optical density treated sample after 72 h; ${OD\;T72}_{c}$ = optical density treated sample. The control was considered PDB+F.

### 4.7. Statistical Analysis

The obtained results from the biological assays were statistically analyzed using an ANOVA test to evaluate the differences between group means with the Statistical Package for the Social Sciences (SPSS), version 13.0 (Prentice Hall, Chicago, IL, USA, 2004). Subsequently, a post hoc Tuckey B test was applied to determine the significance between treatments at *p* < 0.05.

## 5. Conclusions

The current study concluded that the fungal chitosan was extracted from *G. lucidum* with a low M.Wt and moderate viscosity level, and its low degree of deacetylation is in agreement with bibliographic research. It is known that the low M.Wt showed better antimicrobial activity, being easier to penetrate the cell membrane of microbial pathogen. The current study also concluded the usefulness of using fungal-extracted chitosan from *G. lucidum* for its antifungal efficacy against *M fructicola* which causes the brown-rot of apple. As well-known postharvest diseases are difficult to control due to the few available authorized molecules for their control, the treatment with fungal chitosan as an alternative to chemical pesticides can be a useful agent for fruits in postharvest treatment to avoid fungal infection and maintain the quality parameters. In addition, the use of fungal chitosan from *G. lucidum* was able to maintain the quality parameters of treated apples even to 14 DAC due to reducing weight loss and firmness, which can extend the shelf-life of fruits. However, among the possible limitations, some analyses should be considered in future studies to validate the application of fungal chitosan under commercial storage conditions, optimize treatment parameters, and confirm its safety and economic feasibility. Future studies should also clarify the molecular mechanisms involved in chitosan-induced plant defense responses to improve its use as a natural postharvest biocontrol agent.

## Figures and Tables

**Table 1:** Dry weight of fungal biomass, chitin and chitosan extracted from *G. lucidum*.

	Weight (g)	Yield (%)
Fungal fresh mycelium	55.93 ± 5.65 *	
Fungal biomass	11.80 ± 0.88	21.10
Extracted chitin	1.97 ± 0.27	16.69
Obtained chitosan	0.43 ± 0.14	3.64

(*) All values are expressed as mean value of 3 replicates (±SDs).

**Table 2:** FT-IR analysis of F.Cs compared to C.Cs and Ct.

Functional Groups and Vibration Modes	C.Cs	F.Cs	Ct
O-H stretching of OH group	3446 s,br	3450 vs,br	3470 s,br
C-H stretching of CH_2_OH group	2880 m	2883 vw 2386 vw 2068 vw	3110 m 2889 m
ν (C=O) in NHCOCH_3_ group (amide I band)	1653 vs	1640 s	1659 vs
ν (NH_2_) in NHCOCH_3_ group (amide II band)	1572 m	1577 m	1556 m
δ(CH_2_) in CH_2_OH group	1423 m	1432 m	1421 m
δ_s_(CH_3_) in NHCOCH_3_ group	1380 w	1385 m	1374 m
ν_s_(C–O–C) (glycosidic linkage)	1155 m	1150 m	1150 s
C-N stretching	1074 s	1070 m	1075 m
C-C stretching	1023 m	1026 w	1021 s
NH_2_ wagging	870 w	867 w	878 m
O-H out-of-plane bending	535 w	592 m 537 m	556 m 538 m

Keys: s = strong, w = weak, m = medium, br = broad, vs = very strong, vw = very weak, ν = stretching, δ = bending, cm^−1^ is wave number, where (F.Cs) fungal chitosan; (C.Cs) commercial chitosan; (Ct) chitin.

**Table 3:** Physical properties of the studied fungal-derived chitosan.

Parameter	Fungal Chitosan (F.Cs)
Intrinsic Viscosity [η]	1.3–1.6 cP
Molecular Weight (M.Wt)	5.3 × 10^4^ g/mol (53 kDa)
Degree of Deacetylation (DD%)	63.0%

**Table 4:** MIC values of chitosan extracted from *G. lucidum*.

	MIC (mg/mL) of Chitosan Against Tested Fungi		
Samples	*P. expansum*	*M. fructicola*	*B. cinerea*
F.Cs	3.0 _a_	0.75 _a_	0.75 _a_
C.Cs	3.0 _a_	3.0 _b_	1.5 _b_

Data represent the average from three repetitions. Mean values in each column with different letters show statistically significant variation at the *p* < 0.05 level using SPSS software with Tukey’s B test. Abbreviations: F.Cs = chitosan extracted from *G. lucidum*; C.Cs = commercial chitosan; MIC = minimum inhibitory concentration.

**Table 5:** MFC values of chitosan extracted from *G. lucidum*.

	MFC (mg/mL) of Chitosan Against Tested Fungi		
Samples	*P. expansum*	*M. fructicola*	*B. cinerea*
F.Cs	6.0 _a_	0.75 _a_	1.5 _a_
C.Cs	6.0 _a_	6.0 _b_	3.0 _b_

Data represent the average from three repetitions. Mean values in each column with different letters show statistically significant variation at the *p* < 0.05 level using SPSS software with Tukey’s B test. Abbreviations: MFC = minimum fungicidal concentration.

**Table 6:** Infection lesion diameter (mm) of brown-rots disease on apple fruits.

	Lesion Diameter (mm)	
	Prevention	Curative
C-H_2_O	0.0 ± 0 a	0.0 ± 0 a
C+Cyclo.	14.5 ± 0.7 b	17.3 ± 0.9 b
C-Path	63.8 ± 4.0 e	71.7 ± 2.4 f
F.Cs 0.5%	44.3 ± 3.3 d	53.0 ± 2.8 e
F.Cs 1%	13.5 ± 2.1 b	41.2 ± 3.1 d
F.Cs 1.5%	7.3 ± 0.9 b	12.8 ± 1.6 b
C.Cs 0.5%	34.0 ± 1.4 c	57.0 ± 4.2 e
C.Cs 1%	22.5 ± 3.5 c	39.3 ± 3.3 d
C.Cs 1.5%	14.0 ± 1.4 b	27.0 ± 4.2 c

Values followed with different letters for each model are significantly different at *p* < 0.05 using SPSS. Data for each bar are expressed as the mean value of three replicates ± SDs.

**Table 7:** Net protein leakage (ΔA280).

Sample	T_24_	T_48_	T_72_
PDB+F	0.311 ± 0.01 a	0.426 ± 0.0 a	0.473 ± 0.0 a
C.Cs 1.5%	0.384 ± 0.01 ab	0.648 ± 0.0 b	0.933 ± 0.0 b
F.Cs 1.5%	0.484 ± 0.0 c	0.706 ± 0.0 c	0.964 ± 0.01 bc

Values followed by different letters for each column are significantly different at *p* < 0.05. Data are presented as the mean of three replicates ± SD.

**Table 8:** Net DNA leakage (ΔA260).

Sample	T_24_	T_48_	T_72_
PDB+F	0.224 ± 0.01 a	0.436 ± 0.0 a	0.776 ± 0.0 a
C.Cs 1.5%	0.340 ± 0.0 b	0.634 ± 0.01 b	0.873 ± 0.0 b
F.Cs 1.5%	0.357 ± 0.0 bc	0.648 ± 0.01 bc	0.971 ± 0.02 c

Values followed by different letters for each column are significantly different at *p* < 0.05. Data are presented as the mean of three replicates ± SD.

**Table 9:** The disease scale (brown-rot symptoms caused by *M. fructicola*).

Score	Lesion Coverage	Description
N0	0%	no visible symptoms
N1	1–25%	slight decay
N2	26–50%	moderate decay
N3	>50%	severe decay

## Data Availability

The raw data supporting the conclusions of this article will be made available by the authors on request.
